# Spectralis Optical Coherence Tomography for Evaluating Ocular Hypertensive and Glaucoma Suspect Eyes: Real-World Data from Taiwan

**DOI:** 10.3390/diagnostics15101256

**Published:** 2025-05-15

**Authors:** Man-Sze Wong, Chao-Wei Wu, Yue-Cune Chang, Hsin-Yi Chen

**Affiliations:** 1 School of Medicine, College of Medicine, Fu Jen Catholic University, New Taipei City 242062, Taiwan; 408510411@m365.fju.edu.tw; 2 Ophthalmology, Cishan Hospital, Ministry of Health and Welfare, Kaohsiung City 84247, Taiwan; 1050552@ms.kmuh.org.tw; 3 Department of Ophthalmology, Kaohsiung Medical University Hospital, Kaohsiung Medical University, Kaohsiung City 80756, Taiwan; 4 Department of Mathematics, Tamkang University, New Taipei City 25137, Taiwan; ychang@math.tku.edu.tw; 5 Department of Ophthalmology, Fu Jen Catholic University Hospital, New Taipei City 24352, Taiwan; 6 School of Medicine, College of Medicine, Kaohsiung Medical University, Kaohsiung City 80756, Taiwan

**Keywords:** optical coherence tomography, ocular hypertensive, glaucoma suspect, Taiwan

## Abstract

**Objectives**: The aim of this research was to evaluate the diagnostic performance of Spectralis optical coherence tomography (OCT) parameters for ocular hypertensive (OH) and glaucoma suspect (GS) eyes in an Asian population from Taiwan. **Methods**: This retrospective cross-sectional study included 258 OH (mean deviation [MD]: −1.10 ± 1.75 dB), 380 GS (MD: −1.24 ± 2.63 dB), and 742 normal (MD: −1.47 ± 3.29 dB) eyes. The diagnostic performance of Spectralis OCT parameters, including optic nerve head (ONH) and macular parameters, was compared among groups. The area under the receiver operating characteristic curve (AUC) of each parameter signified its power to differentiate between normal and OH or GS eyes. **Results**: In various scanning protocols, circumpapillary retinal nerve fiber layer (NFL)-temporal (AUC = 0.538), macular NFL-outer temporal (AUC = 0.611), and retinal average thickness (RAT)_1.8 (AUC = 0.578) were the best parameters in distinguishing OH eyes from normal eyes. Moreover, minimum rim width (MRW)-mean global (AUC = 0.737), macular NFL-outer temporal (AUC = 0.558), and RAT_2.8 (AUC = 0.543) were the best parameters in distinguishing GS eyes from normal eyes. After adjusting for age and refraction effects, we determined that the AUCs for OH and GS were 0.694 and 0.646, respectively. **Conclusions**: Our real-world data indicate that Spectralis OCT parameters show some potential for early glaucoma detection and monitoring, but their current diagnostic effectiveness remains limited. When managing OH eyes, caution is required in evaluating macular retinal NFL thickness in addition to the ONH. Bruch’s membrane opening–MRW is a potential objective indicator of ONH changes in GS eyes.

## 1. Introduction

Glaucoma is an optic neuropathy characterized by the degeneration of retinal ganglion cells (RGCs), which engenders irreversible damage to the visual field. The loss of vision often goes undetected by patients because glaucoma remains asymptomatic until sufficient sectorial loss of nerve fiber has occurred. Early intervention could minimize such irreversible vision damage and preserve the health of the optic nerve, but diagnosing glaucoma in its early stages remains challenging. One theory suggests that glaucoma progression is due to increased intraocular pressure (IOP), whereas other theories suggest that disease progression is due to vascular and neurodegenerative problems; hence, IOP cannot be a reliable diagnostic parameter for glaucoma [1]. The advent of spectral-domain optical coherence tomography (SD-OCT) systems has enabled clinicians to objectively diagnose and monitor glaucomatous optic neuropathy, especially by focusing on the optic nerve head (ONH), peripapillary retinal nerve fiber layer (ppRNFL), and macular parameters [2,3]. One such system is the Spectralis OCT system (Heidelberg Engineering, Inc., Heidelberg, Germany), a commercially available SD-OCT instrument; this system has been used to calculate Bruch’s membrane opening-based minimum rim width (BMO-MRW) and ppRNFL thickness both globally and locally for certain optic disc sectors [4,5,6]. The Spectralis OCT system is equipped with posterior pole asymmetry analysis software that could increase diagnostic accuracy for glaucoma [7].

Ocular hypertensive (OH) eyes and glaucoma suspect (GS) eyes are risk factors for glaucoma [8,9]. The Ocular Hypertension Treatment Study revealed initial changes in the ONH associated with the onset of glaucoma, including rim thinning, hemorrhage, and abnormal cup-to-disc ratio (CDR) [10]. In addition to exhibiting abnormal cupping, GS eyes have a thinner RNFL [9,11]. However, few studies have reported the performance of Spectralis OCT in examining GS and OH eyes. Moreover, Spectralis OCT lacks a normative database for individuals of Asian ethnicity. Accordingly, the present study explored the clinical utility of Spectralis OCT for assessing OH and GS eyes by evaluating all ONH and macular parameters in an Asian population.

## 2. Materials and Methods

### 2.1. Study Design

This retrospective, cross-sectional study was approved by the Institutional Review Board of Fu Jen Catholic University Hospital (FJUH) (approval numbers FJUH110131 and FUJH112277) and was conducted in accordance with the ethical principles stipulated in the Declaration of Helsinki. Individuals of Chinese ethnicity with healthy, OH, and GS eyes who visited the Department of Ophthalmology at Fu Jen Catholic University Hospital between September 2021 and January 2024 were recruited by an experienced glaucoma specialist (H.-Y.C.) to participate in this study. Informed consent was obtained from all participants.

All participants were subjected to a complete ophthalmic examination that involved visual acuity testing, IOP measurement, gonioscopy, stereoscopic fundus examination, and standard full-threshold automated perimetry (30-2 mode, Humphrey Field Analyzer, model 740; Carl Zeiss Meditec, Inc., Dublin, CA, USA). The inclusion criterion was as follows: having a best-corrected visual acuity of 20/40 Snellen equivalents or better with clear ocular media. The exclusion criteria were as follows: having considerable opacity of the ocular media, having concomitant retinal disease (including uveitis and glaucoma), or having a history of refractive or vitreoretinal surgery. Healthy eyes were used as controls; healthy eyes were defined as those with (1) an IOP level of <21 mm Hg with no history of elevated IOP, (2) a normal optic disc appearance, and (3) no family history of glaucoma or visual field defects. Patients with OH eyes were defined as those with (1) an IOP level of >21 mm Hg and (2) no signs of optic nerve damage or visual field defects. Patients with GS eyes were defined as those with suspicious optic disc appearance such as enlarged disc cupping (a vertical CDR of >0.6) or a CD asymmetry of >0.2 but no definite abnormal findings on perimetry [12]. 

### 2.2. Spectralis OCT Imaging

**Figure 1:**
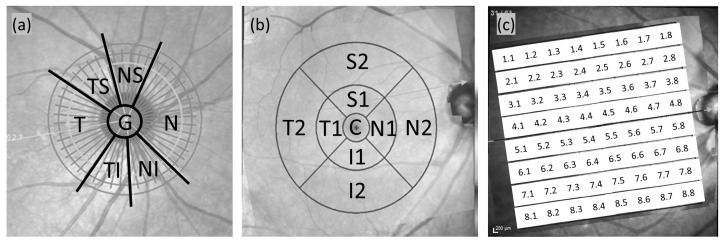
SD-OCT scan labeling of (**a**) six Garway-Heath sections for the circumpapillary retinal fiber layer (cpRNFL) and Bruch’s membrane opening–minimum rim width (BMO-MRW), (**b**) ETDRS grid for retinal thickness, and (**c**) 8 × 8 posterior pole grid for retinal average thickness (RAT). T, temporal; TS, temporal-superior; NS, nasal-superior; N, nasal; NI, nasal-inferior; TI, temporal-inferior; C, central [14].

Information on the ONH and macula was acquired by performing ONH radial and circular scans and posterior pole horizontal scans using a Spectralis OCT system (Figure 1).

To determine the BMO-MRW and thickness of the circumpapillary RNFL, the participants’ optic heads were first located on their respective ONH radial and circular scans. BMO-MRW, the shortest distance between BMO and the internal limiting membrane, was measured through transverse analysis with radial B-scans; the membrane layers were identified automatically using the built-in software. Subsequently, three circular circumpapillary RNFL scans were conducted at different diameters: 3.5, 4.1, and 4.7 mm. Only data from the 3.5 mm diameter scans were analyzed in this study. The results (in μm) are presented as the global average (G) of the measurements and as measurements in six Garway-Heath sectors (Figure 1a) that were established according to their angular degrees around the eye: 315° to 45° (temporal); 45° to 85° (temporal-superior); 85° to 125° (nasal-superior); 125° to 235° (nasal); 235° to 275° (nasal-inferior); and 275° to 315° (temporal-inferior).

To examine the posterior pole, an 8 × 8 grid was placed symmetrically over the fovea–disc axis, followed by a 30° × 25° OCT volume scan of the full-layer retinal thickness. The thickness map of the retinal layers was analyzed by following the posterior pole horizontal scan protocol; specifically, an 8 × 8 grid mode and the Early Treatment Diabetic Retinopathy Study (ETDRS) grid mode were used. In the 8 × 8 grid mode, the scanning area was divided into a total of 64 sections for detailed measurements; the measurements started from the top of the grid moving to the bottom and then from the temporal side to the nasal side (Figure 1c). In the ETDRS grid mode, the scanning area was divided into three concentric rings of different diameters: 1, 3, and 6 mm. The rings were classified into nine sections according to their diameters (Figure 1b). The 1 mm inner ring was used to measure the central thickness (C). The 3 mm intermediate ring was further divided into four quadrants: inner temporal (T1), inner inferior (I1), inner nasal (N1), and inner superior (S1). Similarly, the 6 mm outer ring was divided into four quadrants: outer temporal (T2), outer inferior (I2), outer nasal (N2), and outer superior (S2). All ten retinal layers were segmented automatically, but only the average thicknesses of the full retina, macular NFL, macular ganglion cell layer (GCL), and macular inner plexiform layer (IPL) were analyzed in this study. Because artifacts arising from segmentation errors or poor signals are known to be highly prevalent on circumpapillary RNFL and macular scans [13], raw B-scan images with artifacts were identified and excluded, resulting in the exclusion of data from 5.9% healthy eyes, 9.5% GS eyes, and 11.3% OH eyes.

### 2.3. Statistical Analysis

The baseline characteristics and thickness data of the control group and the OH or GS group were compared using Pearson’s Chi-square test and an independent t test, respectively; for each comparison, only one eye from each participant was randomly selected. The diagnostic performance of different parameters in distinguishing between the groups was determined by comparing their areas under the receiver operating characteristic curves (AUCs). Additionally, 95% confidence intervals (CIs) were computed to evaluate the differences in the performance of the parameters in distinguishing between the healthy control group and the OH or GS group. The likelihood of the parameters to distinguish between the different groups was maximized using a multiple logistic regression model with generalized estimating equations; an “exchangeable” working correlation matrix was used as the parameter-based classifier. All analyses were performed using SPSS software (v.26; IBM Corp., Armonk, NY, USA), and *p* < 0.05 was considered to indicate statistical significance.

## 3. Results

### Demographic and Clinical Data

Table 1 presents the baseline characteristics of all the groups. A total of 742 healthy eyes (mean deviation [MD]: −1.47 ± 3.29 dB; *n* = 393), 258 OH eyes (MD: −1.10 ± 1.75 dB; *n* = 139), and 380 GS eyes (MD: −1.24 ± 2.63 dB; *n* = 208) were included in our study. The control group was significantly older than the OH and GS groups (*p* < 0.001). The groups did not differ significantly in terms of sex or MD.

Table 2 presents a comparison of the thickness and diagnostic performance of the best parameters in the various scan protocols in the OH group. Table S1 provides detailed information on all parameters. Among the ONH parameters, RNFL (temporal) and BMO-MRW (temporal) exhibited fair discriminating power, with their AUCs being 0.538 and 0.535, respectively. In the ETDRS mode, the parameters with the best performance in differentiating OH eyes from normal eyes were nerve fiber layer (NFL)-outer temporal (T2; AUC = 0.611), NFL-outer nasal (N2; AUC = 0.595), whole retina-outer superior (S2; AUC = 0.566), and IPL-outer inferior (I2; AUC = 0.566). In the 8 × 8 grid mode, the parameters with the best performance, as determined in posterior pole asymmetry analysis (64 parameters), were retinal average thickness (RAT) 1.8 (AUC = 0.578), RAT 7.4 (AUC = 0.568), and RAT 8.2 (AUC = 0.568).

Table 3 presents a comparison of the thickness and diagnostic performance of the best parameters in the various scan protocols in the GS group. Table S2 provides detailed information on all parameters. Among the ONH parameters, RNFL (temporal inferior) and BMO-MRW (mean global) exhibited fair discriminating power, with their AUCs being 0.591 and 0.737, respectively. Notably, all BMO-MRW sectors exhibited strong diagnostic performance and a significantly lower thickness in the GS group than in the control group. In the ETDRS mode, the best parameters were whole retina-inner inferior (I1; AUC = 0.520), NFL-outer temporal (T2; AUC = 0.558), GCL-outer superior (S2; AUC = 0.552), and IPL-outer temporal (T2; AUC = 0.544). In the 8 × 8 grid mode, the best parameter was RAT 2.8 (AUC = 0.543), as determined in posterior pole asymmetry analysis (64 parameters).

**Figure 2:**
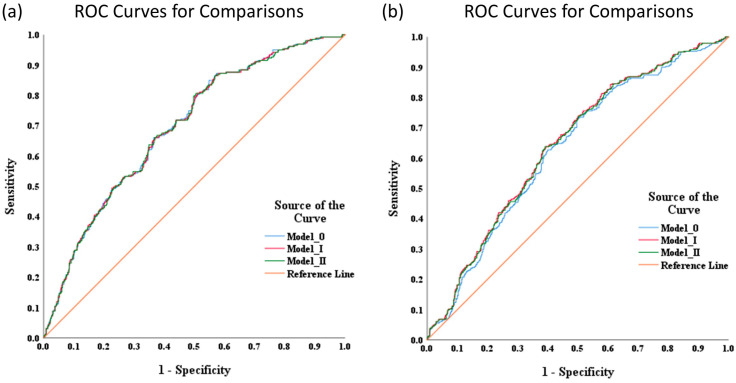
Receiver operating characteristic curves comparing the optimal combinations of parameters for screening individuals (**a**) with OH eyes and (**b**) with GS eyes.

To assess the effects of integrating all parameters, measured through Spectralis OCT with/without adjustment for the effects of confounding variables, the GEE method’s multiple logistic regression analyses were performed with an “exchangeable” working correlation matrix (Figure 2).

Compared with the single parameter NFL_T2 (AUC = 0.611; 95% CI: 0.570–0.653), the integration of age, refraction, and BMO-MRW_T (model 1) resulted in increased diagnostic power for distinguishing OH eyes from normal eyes, with the AUC being 0.694 (0.658–0.730; Table 4). The optimal combination of parameters for distinguishing GS eyes from normal eyes comprised age, refraction, and BMO-MRW_G; the corresponding AUC was 0.646 (0.613–0.679), which was not superior to the AUC value associated with the single parameter BMO-MRW_G (0.737, 95% CI: 0.707–0.767).

## 4. Discussion

SD-OCT overcomes the limitations of time-domain OCT by offering a noninvasive, high-speed method to capture cross-sectional images of ocular structures with adequate axial resolution. Therefore, SD-OCT is more sensitive than time-domain OCT [15] for detecting early RNFL defects and has become a standard diagnostic protocol for macular disease, diabetic retinopathy, and glaucoma [16]. The ONH, ppRNFL, and macula have been traditionally studied to confirm a glaucoma diagnosis [17]. The amount of neuroretinal rim around the ONH can be assessed by determining the CDR (traditional) and BMO-MRW (increased sensitivity) using SD-OCT, both of which serve as early indicators of a risk of glaucoma. The CDR is defined by two landmarks, the optic disc margin and the optic cup margin. The more recently developed Spectralis OCT for measuring BMO-MRW corrects for different neuroretinal rim orientations and defines the true anatomical border of RGC axons. Spectralis OCT is different from other SD-OCT instruments because it provides a unique approach for calculating the BMO-MRW of optic disc sections [4,5,6] and for macula segmentation [18,19]. In our RNFL thickness evaluation, we included data from only 3.5 mm diameter scans (as standard glaucoma application) [20]. The macular region contains large quantities of RGCs. Changes in thickness or the asymmetrical loss of GCLs is the most significant classifier for preperimetric glaucoma [21]. Therefore, this study focused on the segmentation of the three inner layers of the retina, namely, the macular NFL, macular GCL, and macular IPL.

From our current results, the AUC of individual parameters of Spectralis OCT was not high (AUC: 0.5–0.737) in either discriminating OH or GS from normal eyes; it is mandatory to remind the clinicians of the low diagnostic effectiveness in actual clinical applications as well as potential avenues for improvement. To assess the effects of integrating all parameters, measured through Spectralis OCT with/without adjustment for the effects of confounding variables, the GEE method’s multiple logistic regression analyses were performed with an “exchangeable” working correlation matrix. The results show that the AUCs for OH and GS were 0.694 and 0.646, respectively. Since OH or GS eyes are high risk factors for glaucoma, our results reveal that Spectralis OCT parameters show promise in early glaucoma diagnosis and disease monitoring.

Kim et al. reported that segmented IPL thickness was significantly associated with the degree of glaucoma [22]. Through quantitative and qualitative analyses, Lehmann revealed that the thickness of the retinal GCL had similar diagnostic power to RNFL thickness [23]. Moreover, Cifuentes-Canorea et al. demonstrated that the macular inner retinal layers significantly differed between healthy individuals and those with OH eyes as well as between those with OH eyes and patients with early glaucoma [24]. Our results reveal that the best single macular parameter for diagnosing OH eyes was macular NFL (outer temporal section), with the corresponding AUC being 0.611; this finding is consistent with those of Edlinger [25], who reported that structural changes in the temporal sectors of the macular ganglion exhibited comparable performance to peripapillary RNFL thickness in distinguishing between healthy participants those with OH eyes, and those with preperimetric and perimetric glaucoma [24]. Drastic decreases (up to 34%) in retinal thickness at the posterior pole were found to accompany the progression of glaucoma [26]. Measuring retinal thickness at the posterior pole can facilitate the clinical assessment of optic nerve cupping [27]. Salgarello et al. [28] indicated that localized anatomic and functional damage to the inner retina in OH eyes may not develop simultaneously during the early phase of glaucoma. Because the healthy control and OH groups in our study differed significantly in age and refraction, adjusting for the two factors led to an improvement in diagnostic performance, with the AUC value increasing to 0.694. Accordingly, caution should be exercised when evaluating macular RNFL thickness in addition to the ONH during the management of OH eyes.

Studies have revealed that ppRNFL thickness exhibited high diagnostic ability for early glaucoma detection, especially in Caucasian populations [25,29,30]. However, caution should be exercised when diagnosing Asian patients on the basis of only ppRNFL thickness; this is because this parameter was found to vary across races and ethnicities [31]. Patients with GS eyes in this study exhibited significant thinning in the temporal inferior quadrant compared with those with healthy eyes. Conversely, Khanal et al. reported that both the superior RNFL and inferior RNFL were significantly thinner in GS eyes than in normal eyes [32]. We believe that this disparity in findings can be attributed to the different sample sizes and ethnicities of participants in the two studies. Moreover, BMO-MRW (G) was the best single parameter for diagnosing GS eyes in our study (AUC = 0.737), and it substantially outperformed the ppRNFL parameters. This result corroborates the findings of our previous study wherein BMO-MRW (G) yielded the highest AUC and low diagnostic in macular parameters such as RNFL (I1) with 0.520 for discriminating between GS individuals and healthy controls [33]. Yusof et al. also demonstrated that BMO-MRW best distinguishes preperimetric and perimetric glaucomatous eyes from normal eyes [34]. We believe that our findings can be explained by the inclusion criterion for individuals with GS eyes in this study, which was the presence of enlarged cupping.

This study has some limitations. First, because our study was conducted in a real-world clinical setting, imaging data with poor segmentation or alignment errors were excluded [35]. This exclusion may limit the generalizability of the findings, particularly for individuals with high myopia, as their data might have been disproportionately affected. Therefore, our findings should be interpreted with caution, especially for highly myopic individuals. Second, the control group was significantly older than the OH and GS groups (51.77 ± 16.22 years vs. 41.48 ± 16.06 and 45.21 ± 15.29 years). Furthermore, the control group was significantly less myopic than the OH and GS groups (average: −2.31 ± 3.22 D vs. −4.79 ± 3.66 and −3.87 ± 3.50 D). These differences might introduce a bias in data interpretation [31,36,37]. Third, a lack of representative sampling was noted. The study only included patients who visited the ophthalmology department at FJUH. This sample is limited in geographic scope, and the institution represents a specific medical facility type. Therefore, our study sample may not adequately represent the broader population of Taiwan and cannot serve as a representative database for the Asian population. Fourth, this study included only participants of Chinese ethnicity, which may limit the applicability of the findings to the broader Taiwanese population or other ethnic groups. This homogeneity raises concerns about the generalizability of the results to diverse populations. Fifth, systemic disease factors (e.g., diabetes or hypertension), were not evaluated in this retrospective study, which could potentially affect the accuracy of diagnostic performance.

This study has some strengths. Our study, which used real-world data on patients of Asian ethnicity, is among the few studies to use Spectralis OCT for distinguishing GS and OH individuals from healthy controls in a Chinese population in Taiwan. The unique ocular characteristics in this population are challenging for OCT in detecting abnormalities, making these findings particularly relevant and robust. Moreover, this study included a large sample size of 1380 eyes (54% healthy, 19% OH, and 27% GS) and included participants whose ages ranged from 6 to 92 years, which demonstrates that the study sample reflects real-world clinical settings. According to our review of the literature, the Spectralis OCT reference database includes only patients of European descent with ages between 20 and 87 years. Anatomical structures vary across individuals according to their age and race. Caucasian individuals tend to have thinner RNFLs than do Hispanic, Asian [37], and Chinese individuals [38]. Poon et al. demonstrated that both normal aging and ethnicity affect several OCT parameters used to diagnose and monitor glaucoma [31]. We believe that our findings could serve as a valuable benchmark for comparison with findings regarding glaucoma diagnosis in other ethnic groups.

## 5. Conclusions

Our real-world data indicate that Spectralis OCT parameters show some potential for early glaucoma detection and monitoring, but their current diagnostic effectiveness remains limited. When managing OH eyes, caution should be exercised during the evaluation of macular RNFL thickness in addition to the ONH. BMO-MRW is a potential objective indicator of changes in the ONH in GS eyes. Additional large-scale longitudinal studies are warranted to validate these findings.

## Figures and Tables

**Table 1:** Baseline features of the healthy controls and study groups (one randomly selected eye).

Features	Healthy Controls (*n* = 393; 742 Eyes)	OH (*n* = 139; 258 Eyes)		GS (*n* = 208; 380 Eyes)	
	Mean ± SD	Mean ± SD	*p*	Mean ± SD	*p*
Age (years) ^a^	51.77 ± 16.22	41.48 ± 16.06	<0.001	45.21 ± 15.29	<0.001
Sex (male/female) ^b^	149:244	52:87	1.000	99:109	0.024
Refraction (D) ^a^	−2.31 ± 3.22	−4.79 ± 3.66	<0.001	−3.87 ± 3.50	<0.001
MD (dB) ^a^	−1.47 ± 3.29	−1.10 ± 1.75	0.099	−1.24 ± 2.63	0.384
PSD (dB) ^a^	2.60 ± 2.25	2.02 ± 1.26	<0.001	2.11 ± 1.38	0.001

OH: ocular hypertensive eyes; GS: glaucoma suspect eyes; MD: mean deviation; PSD: pattern standard deviation. ^a^: Independent *t* test; ^b^: Pearson’s chi-square test.

**Table 2:** Comparison of thickness and diagnostic performance of the best parameters in different scan protocols in patients with ocular hypertensive eyes.

Scan	Best Parameter	Thickness (µM) (Mean ± SD)		*p* *	AUC (95% CI)	Sensitivity at 95% Specificity (%)	Sensitivity at 80% Specificity (%)
		OH	Control				
RNFL	Temporal (T)	85.14 ± 18.68	84.51 ± 32.73	0.790	0.538 (0.497, 0.580)	4.2	24.7
BMO-MRW	Temporal (T)	222.60 ± 46.31	220.39 ± 53.23	0.681	0.535 (0.495, 0.575)	2.7	20.5
ETDRS							
RETINA	Outer superior (S2)	299.00 ± 16.03	295.30 ± 15.62	0.020	0.566 (0.525, 0.606)	10.4	28.2
NFL	Outer temporal (T2)	19.45 ± 4.74	19.59 ± 2.72	0.657	0.611 (0.570, 0.653)	3.9	21.6
GCL	Outer inferior (I2)	32.42 ± 3.69	32.06 ± 3.93	0.431	0.540 (0.499, 0.582)	8.9	27.0
IPL	Outer inferior (I2)	27.07 ± 3.08	26.58 ± 3.20	0.109	0.566 (0.524, 0.607)	9.3	32.0
PPAA	RAT_18	0.285 ± 0.02	0.292 ± 0.02	0.008	0.578 (0.538, 0.617)	7.4	24.5
	RAT_74	0.295 ± 0.02	0.291 ± 0.02	0.025	0.568 (0.527, 0.609)	9.3	26.3
	RAT_82	0.240 ± 0.01	0.237 ± 0.01	0.013	0.568 (0.526, 0.609)	10.1	28.3

OH: ocular hypertensive eyes; AUC: area under the receiver operating characteristic curve; CI: confidence interval; RNFL: circumpapillary retinal nerve fiber layer; BMO-MRW: Bruch’s membrane opening–minimum rim width; ETDRS: Early Treatment Diabetic Retinopathy Study; RETINA: whole retinal layer; NFL: macular retinal nerve fiber layer; GCL: macular ganglion cell layer; IPL: macular inner plexiform layer; PPAA: posterior pole asymmetry analysis; RAT: retinal average thickness. *: A univariate linear regression model with generalized estimating equations was used along with an “exchangeable” working correlation matrix to account for within-subject dependent variables.

**Table 3:** Comparison of thickness and diagnostic performance of the best parameters in different scan protocols in patients with glaucoma suspect eyes.

Scan	Best Parameter	Thickness (µM) (Mean ± SD)		*p* *	AUC (95% CI)	Sensitivity at 95% Specificity (%)	Sensitivity at 80% Specificity (%)
		GS	Control				
RNFL	Temporal inferior (TI)	152.27 ± 25.05	160.52 ± 30.59	<0.001	0.591 (0.556, 0.626)	8.4	30.7
BMO-MRW	Mean global (G)	249.84 ± 43.88	294.63 ± 58.35	<0.001	0.737 (0.707, 0.767)	17.3	49.6
ETDRS							
RETINA	Inner inferior (I1)	330.78 ± 16.60	332.57 ± 17.31	0.184	0.520 (0.485, 0.555)	7.3	23.6
NFL	Outer temporal (T2)	19.35 ± 3.37	19.59 ± 2.72	0.287	0.558 (0.523, 0.594)	3.4	14.2
GCL	Outer superior (S2)	34.45 ± 3.45	35.16 ± 3.92	0.015	0.552 (0.517, 0.587)	8.9	25.5
IPL	Outer temporal (T2)	31.81 ± 2.74	32.24 ± 2.95	0.054	0.544 (0.509, 0.579)	5.0	29.4
PPAA	RAT_28	0.312 ± 0.02	0.316 ± 0.02	0.074	0.543 (0.507, 0.578)	8.2	24.0

GS: glaucoma suspect eyes; AUC: area under the receiver operating characteristic curve; CI: confidence interval; RNFL: circumpapillary retinal nerve fiber layer; BMO-MRW: Bruch’s membrane opening–minimum rim width; ETDRS: Early Treatment Diabetic Retinopathy Study; RETINA: whole retinal layer; NFL: macular retinal nerve fiber layer; GCL: macular ganglion cell layer; IPL: macular inner plexiform layer; PPAA: posterior pole asymmetry analysis; RAT: retinal average thickness. *: A univariate linear regression model with generalized estimating equations was used along with an “exchangeable” working correlation matrix to account for within-subject dependent variables.

**Table 4:** Optimal combination of parameters for differentiating OH and/or GS eyes from control eyes by using multiple logistic regression with generalized estimating equations and “exchangeable” working correlation matrix.

Subtypes	Parameters Included	AUC (95% CI)	*p*-Value
**OH**	Model 1I: age, refraction, MRW (temporal), RETINA (outer superior)	0.694 (0.658, 0.730)	
	Model 1: age, refraction, MRW (temporal)	0.694 (0.658, 0.730)	
	Model 0: age, refraction	0.694 (0.658, 0.730)	
**GS**	Model II: age, refraction, MRW (mean global), RNFL (temporal inferior)	0.643 (0.609, 0.676)	<0.001 ^b^
	Model I: age, refraction, MRW (mean global)	0.646 (0.613, 0.679)	<0.001 ^a^
	Model 0: age, refraction	0.630 (0.596, 0.664)	

OH: ocular hypertensive eyes; GS: glaucoma suspect eyes; AUC: area under the receiver operating characteristic curve; CI: confidence interval; RNFL: circumpapillary retinal nerve fiber layer; MRW: Bruch’s membrane opening–minimum rim width; NFL: macular retinal nerve fiber layer; GCL: macular ganglion cell layer; IPL: macular inner plexiform layer. MD: mean deviation; PSD: pattern standard deviation. ^a^: Chi-square test for Model 1 versus Model 0. ^b^: Chi-square test for Model II versus Model 0.

## Data Availability

The original contributions presented in this study are included in the article. Further inquiries can be directed to the corresponding author.

## Supplementary Materials

The following supporting information can be downloaded at: https://www.mdpi.com/article/10.3390/diagnostics15101256/s1, Table S1: Comparison of thickness and diagnostic performance of each parameter for each scan in patients with ocular hypertensive eyes; Table S2: Comparison of thickness and diagnostic performance of each parameter for each scan in patients with glaucoma suspect eyes.
