# Supplementary material for: Spectralis Optical Coherence Tomography for Evaluating Ocular Hypertensive and Glaucoma Suspect Eyes: Real-World Data from Taiwan
Source: Diagnostics (Basel). 2025 May 15;15(10):1256. doi: 10.3390/diagnostics15101256 (PMC12110584; doi:10.3390/diagnostics15101256)
Supplement: Supplementary file 1 [file diagnostics-15-01256-s001.zip › Eye, Table S1.pdf]

Table S1. Comparison of Thickness and Diagnostic Performance of Each Parameter for Each Scan in Patients with Ocular Hypertensive Eyes

Scan	Parameter	Thickness (μm) (mean±SD)		*P*	AUC	95% CI	Sensitivity at 95% specificity (%)	Sensitivity at 80% specificity (%)
		**OH**	**Control**					
RNFL	T	85.14 ± 18.68	84.51 ± 32.73	0.790	0.538	0.497, 0.580	4.2	24.7
	TI	159.46 ± 25.42	160.52 ± 30.59	0.467	0.501	0.460, 0.542	2.3	21.6
	NI	113.68 ± 23.12	114.95 ± 29.34	0.494	0.495	0.453, 0.536	4.6	22.4
	N	73.88 ± 18.25	75.05 ± 22.19	0.494	0.495	0.453, 0.537	3.9	22.8
	NS	122.07 ± 25.12	121.45 ± 28.50	0.819	0.516	0.475, 0.557	3.1	22.8
	TS	143.27 ± 25.38	144.59 ± 27.04	0.466	0.494	0.454, 0.535	4.6	19.7
	G	103.51 ± 11.96	103.92 ± 17.54	0.628	0.515	0.474, 0.556	3.1	25.1
MRW	T	222.60 ± 46.31	220.39 ± 53.23	0.681	0.535	0.495, 0.575	2.7	20.5
	TI	320.39 ± 74.72	322.84 ± 69.05	0.574	0.504	0.461, 0.546	5.4	25.5
	NI	346.17 ± 77.85	349.57 ± 72.15	0.491	0.499	0.458, 0.541	5.4	19.7
	N	314.59 ± 77.79	311.80 ± 72.50	0.781	0.526	0.484, 0.568	2.7	23.2
	NS	327.00 ± 75.04	331.55 ± 78.01	0.384	0.491	0.451, 0.532	1.9	19.3
	TS	287.64 ± 68.62	294.47 ± 71.36	0.225	0.483	0.442, 0.524	2.7	20.1
	G	294.33 ± 57.20	294.63 ± 58.35	0.816	0.514	0.473, 0.556	1.2	23.9
ETDRS								
RETINA	T1	323.91 ± 16.03	323.61 ± 16.28	0.857	0.496	0.455, 0.537	6.2	18.5
	T2	280.44 ± 15.57	277.87 ± 14.71	0.136	0.546	0.505, 0.587	8.9	26.6
	I1	333.06 ± 17.32	332.57 ± 17.31	0.968	0.509	0.468, 0.550	5.0	19.7
	I2	283.90 ± 15.52	281.48 ± 16.26	0.237	0.551	0.511, 0.592	6.2	26.6
	N1	337.76 ± 20.27	336.89 ± 17.81	0.902	0.504	0.463, 0.546	5.0	22.4
	N2	315.60 ± 17.42	312.71 ± 18.32	0.156	0.551	0.511, 0.591	6.9	23.9
	S1	337.86 ± 17.51	336.06 ± 16.78	0.419	0.518	0.477, 0.559	7.3	21.6
	S2	299.00 ± 16.03	295.30 ± 15.62	0.020	0.566	0.525, 0.606	10.4	28.2

	C	265.71 ± 23.45	264.55 ± 24.59	0.621	0.510	0.471, 0.550	5.8	20.5
NFL	T1	17.26 ± 1.65	17.51 ± 2.44	0.092	0.534	0.493, 0.575	6.2	33.6
	T2	19.45 ± 4.74	19.59 ± 2.72	0.657	0.611	0.570, 0.653	3.9	21.6
	I1	25.30 ± 3.31	25.58 ± 3.71	0.390	0.524	0.484, 0.565	7.7	29.3
	I2	38.97 ± 6.86	39.94 ± 6.67	0.076	0.546	0.505, 0.588	9.3	29.0
	N1	20.51 ± 2.99	20.82 ± 3.31	0.182	0.536	0.495, 0.577	7.7	37.8
	N2	44.98 ± 8.87	47.74 ± 7.80	< 0.001	0.595	0.555, 0.636	14.7	35.1
	S1	23.94 ± 4.16	24.10 ± 3.34	0.531	0.541	0.500, 0.582	9.7	38.2
	S2	37.75 ± 6.09	38.66 ± 5.58	0.076	0.560	0.518, 0.603	7.3	34.4
	C	11.29 ± 2.63	11.26 ± 2.77	0.885	0.493	0.452, 0.534	12.7	19.7
GCL	T1	46.50 ± 6.36	46.72 ± 6.40	0.344	0.478	0.436, 0.520	6.6	23.9
	T2	34.91 ± 5.29	34.60 ± 4.79	0.620	0.511	0.469, 0.553	6.6	28.6
	I1	50.86 ± 5.50	50.95 ± 5.40	0.704	0.498	0.457, 0.540	4.6	25.9
	I2	32.42 ± 3.69	32.06 ± 3.93	0.431	0.540	0.499, 0.582	8.9	27.0
	N1	48.89 ± 6.47	49.35 ± 5.55	0.317	0.484	0.441, 0.526	7.3	22.0
	N2	39.51 ± 4.07	39.28 ± 4.11	0.707	0.532	0.491, 0.573	3.5	20.5
	S1	51.02 ± 5.59	51.18 ± 5.45	0.685	0.482	0.440, 0.524	8.1	26.3
	S2	35.29 ± 3.98	35.16 ± 3.92	0.704	0.514	0.472, 0.556	6.9	29.0
	C	14.16 ± 4.97	13.85 ± 5.06	0.493	0.524	0.483, 0.565	8.9	29.7
IPL	T1	40.67 ± 3.65	41.18 ± 3.84	0.097	0.458	0.416, 0.499	5.4	21.6
	T2	32.27 ± 3.31	32.24 ± 2.95	0.987	0.504	0.462, 0.547	9.3	25.5
	I1	40.47 ± 3.72	40.52 ± 3.55	0.782	0.501	0.459, 0.542	6.9	27.8
	I2	27.07 ± 3.08	26.58 ± 3.20	0.109	0.566	0.524, 0.607	9.3	32.0
	N1	41.38 ± 4.07	41.61 ± 3.57	0.398	0.482	0.439, 0.524	10.0	27.8
	N2	31.30 ± 3.13	30.76 ± 3.00	0.083	0.561	0.520, 0.601	4.6	35.1
	S1	40.61 ± 3.57	40.72 ± 3.55	0.667	0.488	0.446, 0.530	5.4	30.1
	S2	29.02 ± 2.93	28.74 ± 2.89	0.320	0.545	0.503, 0.586	8.9	30.5
	C	19.30 ± 4.06	19.19 ± 4.16	0.761	0.509	0.469, 0.549	6.9	20.5
PPAA	RAT_11	0.23 ± 0.02	0.23 ± 0.02	0.006	0.556	0.515, 0.597	8.9	25.7

RAT_12	0.24 ± 0.02	0.24 ± 0.01	0.045	0.556	0.515, 0.596	8.6	24.5
RAT_13	0.25 ± 0.02	0.25 ± 0.01	0.150	0.549	0.509, 0.589	6.2	22.2
RAT_14	0.26 ± 0.02	0.26 ± 0.02	0.332	0.545	0.505, 0.585	6.2	23.0
RAT_15	0.27 ± 0.02	0.27 ± 0.02	0.971	0.519	0.479, 0.560	3.9	19.1
RAT_16	0.28 ± 0.02	0.28 ± 0.02	0.337	0.489	0.448, 0.530	3.9	15.6
RAT_17	0.30 ± 0.02	0.29 ± 0.02	0.598	0.526	0.484, 0.567	7.4	22.2
RAT_18	0.29 ± 0.02	0.29 ± 0.02	0.008	0.578	0.538, 0.617	7.4	24.5
RAT_21	0.23 ± 0.01	0.23 ± 0.01	0.094	0.546	0.505, 0.588	10.5	28.3
RAT_22	0.25 ± 0.01	0.24 ± 0.01	0.031	0.555	0.514, 0.595	10.1	27.5
RAT_23	0.27 ± 0.02	0.26 ± 0.01	0.056	0.556	0.516, 0.597	9.3	27.5
RAT_24	0.28 ± 0.02	0.28 ± 0.02	0.139	0.551	0.510, 0.592	5.4	28.3
RAT_25	0.29 ± 0.02	0.29 ± 0.02	0.158	0.554	0.513, 0.594	5.0	31.0
RAT_26	0.29 ± 0.02	0.29 ± 0.02	0.493	0.536	0.496, 0.577	5.0	23.6
RAT_27	0.30 ± 0.02	0.30 ± 0.02	0.586	0.499	0.459, 0.539	5.4	18.6
RAT_28	0.32 ± 0.03	0.32 ± 0.02	0.560	0.495	0.454, 0.536	6.2	16.7
RAT_31	0.24 ± 0.01	0.24 ± 0.01	0.183	0.536	0.495, 0.577	8.9	26.0
RAT_32	0.27 ± 0.02	0.27 ± 0.01	0.157	0.539	0.498, 0.580	10.1	28.3
RAT_33	0.30 ± 0.02	0.30 ± 0.02	0.200	0.539	0.498, 0.579	8.5	24.4
RAT_34	0.33 ± 0.02	0.33 ± 0.02	0.496	0.525	0.484, 0.565	6.6	21.3
RAT_35	0.33 ± 0.02	0.33 ± 0.02	0.449	0.528	0.487, 0.568	6.2	21.3
RAT_36	0.32 ± 0.02	0.32 ± 0.02	0.241	0.544	0.503, 0.584	6.2	23.6
RAT_37	0.31 ± 0.02	0.30 ± 0.02	0.334	0.543	0.503, 0.583	5.4	22.1
RAT_38	0.31 ± 0.03	0.31 ± 0.03	0.326	0.484	0.444, 0.525	5.0	16.7
RAT_41	0.25 ± 0.01	0.25 ± 0.02	0.410	0.531	0.491, 0.572	6.2	24.9
RAT_42	0.29 ± 0.02	0.28 ± 0.02	0.261	0.535	0.494, 0.576	6.6	24.9
RAT_43	0.32 ± 0.02	0.32 ± 0.02	0.816	0.514	0.473, 0.554	6.2	20.6
RAT_44	0.31 ± 0.02	0.31 ± 0.02	0.906	0.493	0.452, 0.533	4.3	20.2
RAT_45	0.31 ± 0.02	0.31 ± 0.02	0.857	0.505	0.465, 0.546	4.3	17.9
RAT_46	0.34 ± 0.02	0.34 ± 0.02	0.977	0.506	0.465, 0.547	5.1	19.5
RAT_47	0.32 ± 0.02	0.32 ± 0.02	0.267	0.543	0.503, 0.584	6.6	25.3

RAT_48	0.30 ± 0.02	0.29 ± 0.02	0.467	0.536	0.495, 0.577	5.4	25.3
RAT_51	0.25 ± 0.01	0.25 ± 0.01	0.021	0.565	0.525, 0.606	8.6	28.0
RAT_52	0.28 ± 0.02	0.28 ± 0.02	0.102	0.559	0.519, 0.599	8.6	26.1
RAT_53	0.32 ± 0.02	0.32 ± 0.02	0.582	0.526	0.485, 0.566	6.6	21.0
RAT_54	0.31 ± 0.02	0.31 ± 0.02	0.800	0.489	0.447, 0.530	6.2	20.6
RAT_55	0.31 ± 0.02	0.31 ± 0.02	0.998	0.493	0.452, 0.533	6.6	19.5
RAT_56	0.34 ± 0.02	0.34 ± 0.02	0.485	0.522	0.481, 0.563	5.1	20.6
RAT_57	0.32 ± 0.02	0.32 ± 0.02	0.158	0.554	0.513, 0.594	5.8	26.8
RAT_58	0.30 ± 0.02	0.30 ± 0.02	0.121	0.551	0.509, 0.592	7.4	30.0
RAT_61	0.25 ± 0.01	0.24 ± 0.01	0.053	0.554	0.514, 0.595	6.6	26.8
RAT_62	0.27 ± 0.01	0.27 ± 0.01	0.018	0.567	0.526, 0.607	10.1	25.7
RAT_63	0.30 ± 0.02	0.30 ± 0.02	0.040	0.561	0.521, 0.601	8.6	26.5
RAT_64	0.33 ± 0.02	0.33 ± 0.02	0.131	0.545	0.504, 0.585	7.4	23.7
RAT_65	0.34 ± 0.02	0.34 ± 0.02	0.147	0.544	0.503, 0.584	7.8	24.9
RAT_66	0.33 ± 0.02	0.33 ± 0.02	0.080	0.555	0.515, 0.595	7.4	26.1
RAT_67	0.31 ± 0.02	0.31 ± 0.02	0.123	0.549	0.509, 0.589	7.4	24.5
RAT_68	0.31 ± 0.02	0.31 ± 0.02	0.689	0.520	0.479, 0.561	8.2	20.2
RAT_71	0.24 ± 0.01	0.23 ± 0.01	0.050	0.560	0.519, 0.600	8.9	26.6
RAT_72	0.25 ± 0.01	0.25 ± 0.01	0.035	0.563	0.522, 0.604	10.0	28.2
RAT_73	0.27 ± 0.01	0.27 ± 0.01	0.033	0.565	0.524, 0.605	9.7	27.8
RAT_74	0.30 ± 0.02	0.29 ± 0.02	0.025	0.568	0.527, 0.609	9.3	26.3
RAT_75	0.31 ± 0.02	0.30 ± 0.02	0.027	0.566	0.525, 0.606	10.0	26.6
RAT_76	0.30 ± 0.02	0.30 ± 0.02	0.047	0.559	0.519, 0.599	10.8	25.9
RAT_77	0.30 ± 0.02	0.30 ± 0.02	0.331	0.528	0.488, 0.568	7.7	22.0
RAT_78	0.31 ± 0.02	0.31 ± 0.02	0.612	0.486	0.445, 0.526	5.4	17.0
RAT_81	0.23 ± 0.01	0.23 ± 0.01	0.052	0.555	0.514, 0.595	7.8	30.6
RAT_82	0.24 ± 0.01	0.24 ± 0.01	0.013	0.568	0.526, 0.609	10.1	28.3
RAT_83	0.25 ± 0.01	0.25 ± 0.01	0.048	0.550	0.509, 0.591	9.3	28.7
RAT_84	0.27 ± 0.01	0.26 ± 0.02	0.139	0.553	0.513, 0.593	7.8	24.8
RAT_85	0.28 ± 0.02	0.27 ± 0.01	0.034	0.560	0.520, 0.601	8.1	28.7

RAT_86	0.28 ± 0.02	0.28 ± 0.02	0.185	0.538	0.498, 0.578	6.2	24.4
RAT_87	0.29 ± 0.02	0.29 ± 0.02	0.867	0.502	0.462, 0.542	5.0	18.2
RAT_88	0.30 ± 0.02	0.30 ± 0.02	0.684	0.503	0.462, 0.544	5.4	21.3

OH: ocular hypertensive eyes; AUC: area under the receiver operating characteristic curve; CI: confidence interval; RNFL: circumpapillary retinal nerve fiber layer; MRW: Bruch's membrane opening-minimum rim width; ETDRS: Early Treatment Diabetic Retinopathy Study; PPAA: posterior pole asymmetry analysis; RETINA: whole retinal layer; NFL: macular retinal nerve fiber layer; GCL: macular ganglion cell layer; IPL: macular inner plexiform layer; RAT: retinal average thickness; T: temporal; TI: temporal inferior; NI: nasal inferior; N: nasal; NS: nasal superior; TS: temporal superior; G: global; T1: inner temporal; T2: outer temporal; I1: inner inferior; I2: outer inferior; N1: inner nasal; N2: outer nasal; S1: inner superior; S2: outer superior; C: central

* Bold *P* values are significant
