# Supplementary material for: Spectralis Optical Coherence Tomography for Evaluating Ocular Hypertensive and Glaucoma Suspect Eyes: Real-World Data from Taiwan
Source: Diagnostics (Basel). 2025 May 15;15(10):1256. doi: 10.3390/diagnostics15101256 (PMC12110584; doi:10.3390/diagnostics15101256)
Supplement: Supplementary file 1 [file diagnostics-15-01256-s001.zip › Eye, Table S2.pdf]

Table S2. Comparison of Thickness and Diagnostic Performance of Each Parameter for Each Scan in Patients with Glaucoma Suspect Eyes

Scan	Parameter	Thickness (μm) (mean±SD)		*P*	AUC	95% CI	Sensitivity at 95% specificity (%)	Sensitivity at 80% specificity (%)
		**GS**	**Control**					
RNFL	T	80.32 ± 15.27	84.51 ± 32.73	0.009	0.534	0.498, 0.569	9.2	21.5
	TI	152.27 ± 25.05	160.52 ± 30.59	< 0.001	0.591	0.556, 0.626	8.4	30.7
	NI	110.98 ± 25.23	114.95 ± 29.33	0.047	0.541	0.505, 0.578	11.3	29.1
	N	75.43 ± 15.98	75.05 ± 22.19	0.796	0.482	0.447, 0.518	5.0	19.7
	NS	122.25 ± 32.90	121.45 ± 28.50	0.764	0.493	0.457, 0.529	5.5	22.3
	TS	141.34 ± 24.62	144.59 ± 27.04	0.089	0.529	0.494, 0.564	8.4	22.8
	G	101.49 ± 11.93	103.92 ± 17.54	0.021	0.546	0.510, 0.582	8.4	28.1
MRW	T	194.19 ± 42.60	220.39 ± 53.23	< 0.001	0.642	0.608, 0.675	13.6	36.7
	TI	272.28 ± 61.35	322.84 ± 69.05	< 0.001	0.716	0.685, 0.747	16.8	46.2
	NI	298.82 ± 63.36	349.57 ± 72.15	< 0.001	0.710	0.679, 0.741	14.7	48.3
	N	258.11 ± 63.49	311.80 ± 72.50	< 0.001	0.716	0.684, 0.747	18.6	51.7
	NS	279.83 ± 64.67	331.55 ± 78.01	< 0.001	0.703	0.672, 0.734	13.1	44.4
	TS	249.40 ± 58.37	294.47 ± 71.36	< 0.001	0.694	0.662, 0.725	11.8	44.4
	G	249.84 ± 43.88	294.63 ± 58.35	< 0.001	0.737	0.707, 0.767	17.3	49.6
ETDRS								
RETINA	T1	322.56 ± 15.46	323.61 ± 16.28	0.377	0.512	0.477, 0.547	2.9	15.0
	T2	278.06 ± 13.92	277.87 ± 14.71	0.987	0.490	0.455, 0.525	4.7	17.8
	I1	330.78 ± 16.60	332.57 ± 17.31	0.184	0.520	0.485, 0.555	7.3	23.6
	I2	280.37 ± 13.99	281.48 ± 16.26	0.281	0.508	0.473, 0.543	1.8	18.4
	N1	335.36 ± 17.90	336.89 ± 17.81	0.278	0.517	0.482, 0.553	2.9	17.8
	N2	312.34 ± 15.88	312.71 ± 18.32	0.754	0.497	0.461, 0.532	1.8	21.0
	S1	334.93 ± 16.43	336.06 ± 16.78	0.400	0.512	0.476, 0.547	3.4	17.1
	S2	295.71 ± 14.21	295.30 ± 15.62	0.772	0.487	0.451, 0.523	4.7	20.2

	C	265.21 ± 23.63	264.55± 24.59	0.618	0.491	0.455, 0.526	4.2	24.1
NFL	T1	17.24 ± 1.41	17.51 ± 2.44	0.027	0.532	0.498, 0.567	4.7	30.2
	T2	19.35 ± 3.37	19.59 ± 2.72	0.287	0.558	0.523, 0.594	3.4	14.2
	I1	25.42 ± 3.91	25.58 ± 3.71	0.656	0.502	0.466, 0.538	11.5	27.6
	I2	39.09 ± 6.64	39.94 ± 6.67	0.102	0.524	0.489, 0.560	10.8	24.7
	N1	20.67 ± 3.25	20.82 ± 3.31	0.499	0.520	0.484, 0.556	6.0	16.5
	N2	46.13 ± 7.64	47.74 ± 7.80	0.004	0.548	0.513, 0.584	12.1	26.0
	S1	23.85 ± 3.33	24.10 ± 3.34	0.286	0.518	0.483, 0.554	10.2	32.5
	S2	38.19 ± 5.78	38.66 ± 5.58	0.241	0.522	0.486, 0.558	6.1	23.9
	C	11.24 ± 2.88	11.26 ± 2.77	0.966	0.503	0.467, 0.538	10.5	22.3
GCL	T1	46.13 ± 6.18	46.72 ± 6.40	0.222	0.522	0.487, 0.557	7.1	25.2
	T2	34.10 ± 4.50	34.60 ± 4.79	0.173	0.535	0.500, 0.570	6.3	24.7
	I1	50.09 ± 5.41	50.95 ± 5.40	0.057	0.538	0.503, 0.573	7.1	27.6
	I2	31.47 ± 3.57	32.06 ± 3.93	0.034	0.549	0.513, 0.584	6.6	26.5
	N1	48.48 ± 6.32	49.35 ± 5.55	0.056	0.526	0.490, 0.561	7.3	22.8
	N2	38.80 ± 3.83	39.28 ± 4.11	0.132	0.529	0.494, 0.564	5.2	26.5
	S1	50.39 ± 5.60	51.18 ± 5.45	0.078	0.534	0.499, 0.570	6.0	27.3
	S2	34.45 ± 3.45	35.16 ± 3.92	0.015	0.552	0.517, 0.587	8.9	25.5
	C	14.16 ± 5.20	13.85 ± 5.06	0.379	0.475	0.439, 0.510	9.2	16.0
IPL	T1	40.77 ± 3.69	41.18 ± 3.84	0.181	0.526	0.491, 0.562	8.9	28.9
	T2	31.81 ± 2.74	32.24 ± 2.95	0.054	0.544	0.509, 0.579	5.0	29.4
	I1	40.07 ± 3.51	40.52 ± 3.55	0.137	0.538	0.502, 0.563	7.6	26.8
	I2	26.24 ± 2.79	26.58 ± 3.20	0.106	0.528	0.493, 0.564	7.9	23.9
	N1	41.21 ± 4.16	41.61 ± 3.57	0.194	0.528	0.492, 0.564	8.1	29.1
	N2	30.51 ± 2.95	30.76 ± 3.00	0.367	0.524	0.489, 0.560	7.6	24.1
	S1	40.28 ± 3.85	40.72 ± 3.55	0.125	0.526	0.491, 0.561	8.1	24.4
	S2	28.40 ± 2.85	28.74 ± 2.89	0.161	0.533	0.497, 0.568	8.1	22.8
	C	19.02 ± 3.92	19.19 ± 4.16	0.643	0.505	0.469, 0.540	6.6	24.4
PPAA	RAT_11	0.23 ± 0.01	0.23 ± 0.02	0.869	0.506	0.471, 0.542	3.7	19.0
	RAT_12	0.23 ± 0.01	0.24 ± 0.01	0.329	0.519	0.484, 0.554	4.7	20.1

RAT_13	0.25 ± 0.01	0.25 ± 0.01	0.238	0.520	0.485, 0.555	4.7	20.3
RAT_14	0.26 ± 0.01	0.26 ± 0.02	0.265	0.518	0.483, 0.553	4.2	18.7
RAT_15	0.27 ± 0.02	0.27 ± 0.02	0.190	0.522	0.487, 0.557	4.2	24.3
RAT_16	0.28 ± 0.02	0.28 ± 0.02	0.109	0.533	0.498, 0.569	4.7	24.3
RAT_17	0.29 ± 0.02	0.29 ± 0.02	0.231	0.530	0.494, 0.566	4.5	25.3
RAT_18	0.28 ± 0.02	0.29 ± 0.02	0.446	0.530	0.495, 0.565	2.9	22.7
RAT_21	0.23 ± 0.01	0.23 ± 0.01	0.780	0.501	0.465, 0.536	5.0	19.8
RAT_22	0.24 ± 0.01	0.24 ± 0.01	0.701	0.503	0.468, 0.538	2.6	18.2
RAT_23	0.26 ± 0.01	0.26 ± 0.01	0.767	0.497	0.461, 0.532	4.2	19.3
RAT_24	0.28 ± 0.01	0.28 ± 0.02	0.385	0.509	0.473, 0.544	5.8	23.2
RAT_25	0.29 ± 0.02	0.29 ± 0.02	0.273	0.511	0.475, 0.547	6.3	24.3
RAT_26	0.29 ± 0.02	0.29 ± 0.02	0.191	0.513	0.478, 0.548	4.7	22.2
RAT_27	0.30 ± 0.02	0.30 ± 0.02	0.065	0.527	0.492, 0.562	6.1	20.8
RAT_28	0.31 ± 0.02	0.32 ± 0.02	0.074	0.543	0.507, 0.578	8.2	24.0
RAT_31	0.24 ± 0.01	0.24 ± 0.01	0.725	0.498	0.463, 0.533	2.4	18.9
RAT_32	0.27 ± 0.01	0.27 ± 0.01	0.603	0.504	0.469, 0.539	4.2	18.1
RAT_33	0.30 ± 0.02	0.30 ± 0.02	0.287	0.516	0.481, 0.551	5.0	17.8
RAT_34	0.32 ± 0.02	0.33 ± 0.02	0.155	0.522	0.487, 0.557	6.0	21.8
RAT_35	0.33 ± 0.02	0.33 ± 0.02	0.432	0.514	0.478, 0.549	6.0	23.4
RAT_36	0.32 ± 0.02	0.32 ± 0.02	0.794	0.500	0.465, 0.536	4.7	20.5
RAT_37	0.30 ± 0.02	0.30 ± 0.02	0.775	0.496	0.461, 0.532	4.5	18.6
RAT_38	0.31 ± 0.02	0.31 ± 0.03	0.177	0.519	0.484, 0.554	3.9	21.8
RAT_41	0.25 ± 0.01	0.25 ± 0.02	0.487	0.503	0.468, 0.538	4.5	21.4
RAT_42	0.28 ± 0.01	0.28 ± 0.02	0.441	0.513	0.478, 0.548	6.1	20.6
RAT_43	0.32 ± 0.02	0.32 ± 0.02	0.172	0.522	0.487, 0.557	5.8	22.4
RAT_44	0.31 ± 0.02	0.31 ± 0.02	0.624	0.512	0.476, 0.547	5.0	21.4
RAT_45	0.31 ± 0.02	0.31 ± 0.02	0.576	0.511	0.475, 0.546	5.5	19.3
RAT_46	0.34 ± 0.02	0.34 ± 0.02	0.313	0.516	0.480, 0.551	5.8	21.6
RAT_47	0.32 ± 0.02	0.32 ± 0.02	0.895	0.493	0.457, 0.529	4.5	21.6
RAT_48	0.30 ± 0.02	0.29 ± 0.02	0.861	0.487	0.452, 0.523	5.5	18.7

RAT_51	0.25 ± 0.01	0.25 ± 0.01	0.218	0.470	0.435, 0.506	5.3	18.7
RAT_52	0.28 ± 0.02	0.28 ± 0.02	0.772	0.490	0.455, 0.526	7.1	18.4
RAT_53	0.32 ± 0.02	0.32 ± 0.02	0.557	0.501	0.466, 0.537	7.9	20.5
RAT_54	0.31 ± 0.02	0.31 ± 0.02	0.855	0.506	0.470, 0.542	4.5	23.7
RAT_55	0.31 ± 0.02	0.31 ± 0.02	0.491	0.516	0.480, 0.551	6.3	20.3
RAT_56	0.34 ± 0.02	0.34 ± 0.02	0.394	0.514	0.478, 0.550	7.4	21.3
RAT_57	0.32 ± 0.02	0.32 ± 0.02	0.798	0.498	0.463, 0.534	5.5	21.3
RAT_58	0.30 ± 0.02	0.30 ± 0.02	0.587	0.506	0.470, 0.541	6.8	21.3
RAT_61	0.25 ± 0.01	0.24 ± 0.01	0.206	0.473	0.438, 0.508	2.9	20.7
RAT_62	0.27 ± 0.01	0.27 ± 0.01	0.251	0.470	0.435, 0.505	3.7	17.3
RAT_63	0.30 ± 0.02	0.30 ± 0.02	0.995	0.490	0.455, 0.526	4.5	21.3
RAT_64	0.33 ± 0.02	0.33 ± 0.02	0.633	0.503	0.467, 0.538	6.0	22.6
RAT_65	0.34 ± 0.02	0.34 ± 0.02	0.307	0.517	0.482, 0.553	7.9	23.1
RAT_66	0.33 ± 0.02	0.33 ± 0.02	0.342	0.518	0.482, 0.554	7.9	25.2
RAT_67	0.31 ± 0.02	0.31 ± 0.02	0.598	0.503	0.468, 0.539	6.3	21.3
RAT_68	0.31 ± 0.02	0.31 ± 0.02	0.322	0.508	0.473, 0.543	6.8	19.4
RAT_71	0.23 ± 0.01	0.23 ± 0.01	0.406	0.525	0.489, 0.560	3.7	16.0
RAT_72	0.25 ± 0.01	0.25 ± 0.01	0.281	0.535	0.499, 0.570	5.0	17.1
RAT_73	0.27 ± 0.01	0.27 ± 0.01	0.528	0.527	0.491, 0.562	5.2	17.1
RAT_74	0.29 ± 0.01	0.29 ± 0.02	0.692	0.520	0.484, 0.555	4.2	21.8
RAT_75	0.30 ± 0.02	0.30 ± 0.02	0.926	0.513	0.477, 0.549	4.5	20.7
RAT_76	0.30 ± 0.02	0.30 ± 0.02	0.871	0.502	0.466, 0.538	6.3	19.9
RAT_77	0.30 ± 0.02	0.30 ± 0.02	0.515	0.495	0.460, 0.530	5.2	20.2
RAT_78	0.31 ± 0.02	0.31 ± 0.02	0.512	0.499	0.464, 0.534	4.7	20.7
RAT_81	0.23 ± 0.01	0.23 ± 0.01	0.881	0.525	0.470, 0.541	2.9	22.2
RAT_82	0.24 ± 0.01	0.24 ± 0.01	0.132	0.539	0.503, 0.574	4.2	23.2
RAT_83	0.25 ± 0.01	0.25 ± 0.01	0.161	0.535	0.499, 0.570	4.2	25.1
RAT_84	0.26 ± 0.01	0.26 ± 0.02	0.612	0.528	0.492, 0.563	5.0	21.9
RAT_85	0.28 ± 0.01	0.27 ± 0.01	0.391	0.526	0.490, 0.56	5.3	22.2
RAT_86	0.28 ± 0.01	0.28 ± 0.02	0.880	0.509	0.473, 0.544	2.6	20.6

RAT_87	0.29 ± 0.02	0.29 ± 0.02	0.530	0.494	0.459, 0.530	3.2	17.4
RAT_88	0.30 ± 0.02	0.30 ± 0.02	0.657	0.490	0.455, 0.526	3.2	20.3

GS: glaucoma suspect eyes; AUC: area under the receiver operating characteristic curve; CI: confidence interval; RNFL: circumpapillary retinal nerve fiber layer; MRW: Bruch's membrane opening-minimum rim width; ETDRS: Early Treatment Diabetic Retinopathy Study; PPAA: posterior pole asymmetry analysis; RETINA: whole retinal layer; NFL: macular retinal nerve fiber layer; GCL: macular ganglion cell layer; IPL: macular inner plexiform layer; RAT: retinal average thickness; T: temporal; TI: temporal inferior; NI: nasal inferior; N: nasal; NS: nasal superior; TS: temporal superior; G: global; T1: inner temporal; T2: outer temporal; I1: inner inferior; I2: outer inferior; N1: inner nasal; N2: outer nasal; S1: inner superior; S2: outer superior; C: central

* Bold *P* values are significant
